# Supplementary material for: Rhytidhylides A and B, Two New Phthalide Derivatives from the Endophytic Fungus Rhytidhysteron sp. BZM-9
Source: Molecules. 2021 Oct 9;26(20):6092. doi: 10.3390/molecules26206092 (PMC8540479; doi:10.3390/molecules26206092)
Supplement: Supplementary file 1 [file molecules-26-06092-s001.zip › molecules-1370597-supplementary.pdf]

Supplementary Material

Rhytidhylide A and B, Two New Phthalide Derivatives from the Endophytic Fungus *Rhytidhysteron* sp. BZM-9

Sha Zhang [1,2,†], Dekun Chen [1,2,†], Min Kuang [1,2], Weiwei Peng [1,2], Yan Chen [1,2], Jianbing Tan [1,2], Fenghua Kang [1,2], Kangping Xu [1,2], Zhenxing Zou [1,2,*]

[1] Xiangya School of Pharmaceutical Sciences, Central South University, Changsha 410013, China; ad8879789@163.com (S.Z.); 197211019@csu.edu.cn (D.C.); kuangmin@csu.edu.cn (M.K.); pww199802@163.com (W.P.); chenyanya234@163.com (Y.C.); tanjb1009@csu.edu.cn (J.T.); kangfenghua@csu.edu.cn (F.K.); xukp395@csu.edu.cn (K.X.)
[2] Hunan Key Laboratory of Diagnostic and Therapeutic Drug Research for Chronic Diseases, Changsha 410013, China

[*] Correspondence: zouzhenxing@csu.edu.cn (Z.Z.); Tel.: +86-731-82650395 (Z.Z.)
[†] These authors contributed equally to this work.

List of Contents .... Page

Figure S1. HRESIMS spectrum of compound **1** 3

Figure S2. ^{1}H NMR spectrum of compound **1** 3

Figure S3. ^{13}C NMR spectrum of compound **1** 4

Figure S4. ^{1}H-^{1}H COSY spectrum of compound **1** 4

Figure S5. HSQC spectrum of compound **1** 5

Figure S6. HMBC spectrum of compound **1** 5

Figure S7. NOESY spectrum of compound **1** 6

Figure S8. HRESIMS spectrum of compound **2** 6

Figure S9. ^{1}H NMR spectrum of compound **2** 7

Figure S10. ^{13}C NMR spectrum of compound **2** 7

Figure S11. ^{1}H-^{1}H COSY spectrum of compound **2** 8

Figure S12. HSQC spectrum of compound **2** 8

Figure S13. HMBC spectrum of compound **2** 9

Figure S14. NOESY spectrum of compound **2** 9

Figure S15. Antimicrobial activity assay results 10

Figure S16. Dose-response curves for human hepatoma cell lines (HepG2 and SMMC7721) 10

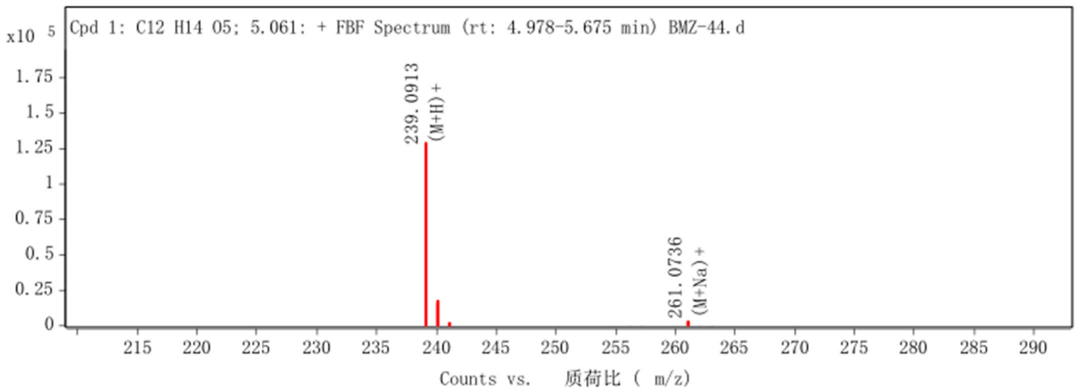

Figure S1. HRESIMS spectrum of compound **1**.

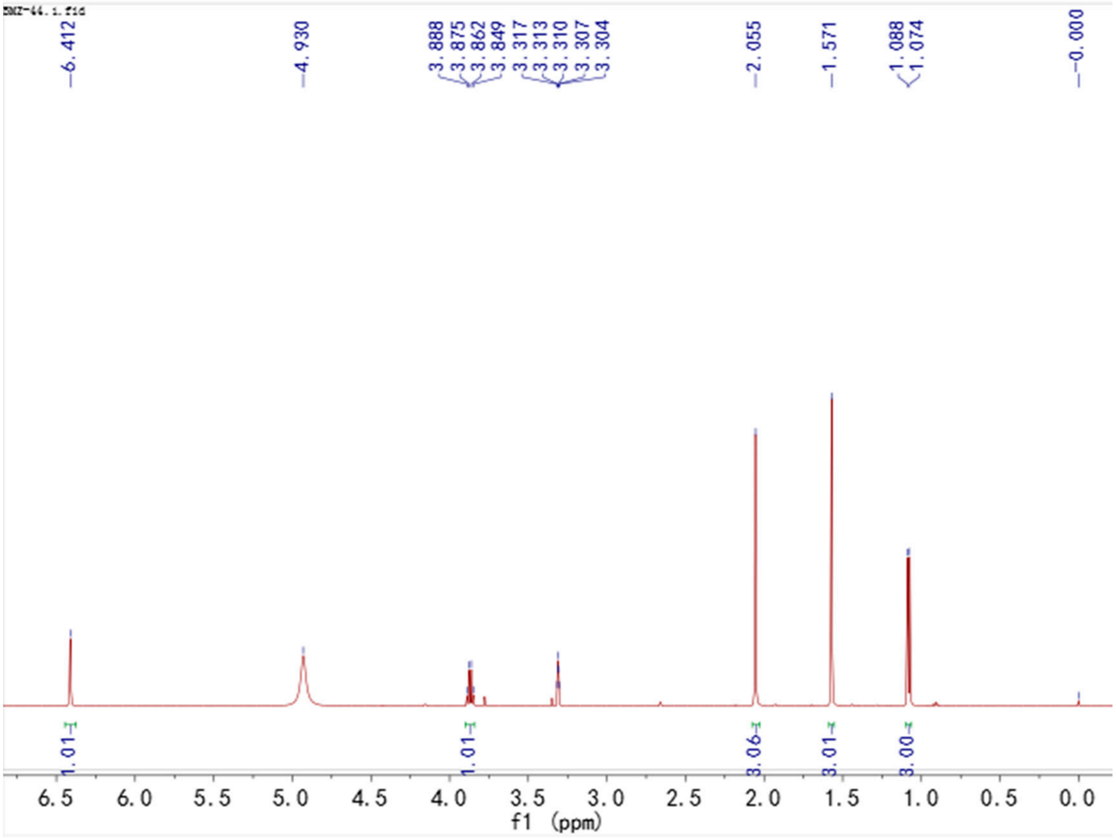

Figure S2. ^{1}H NMR spectrum of compound **1**.

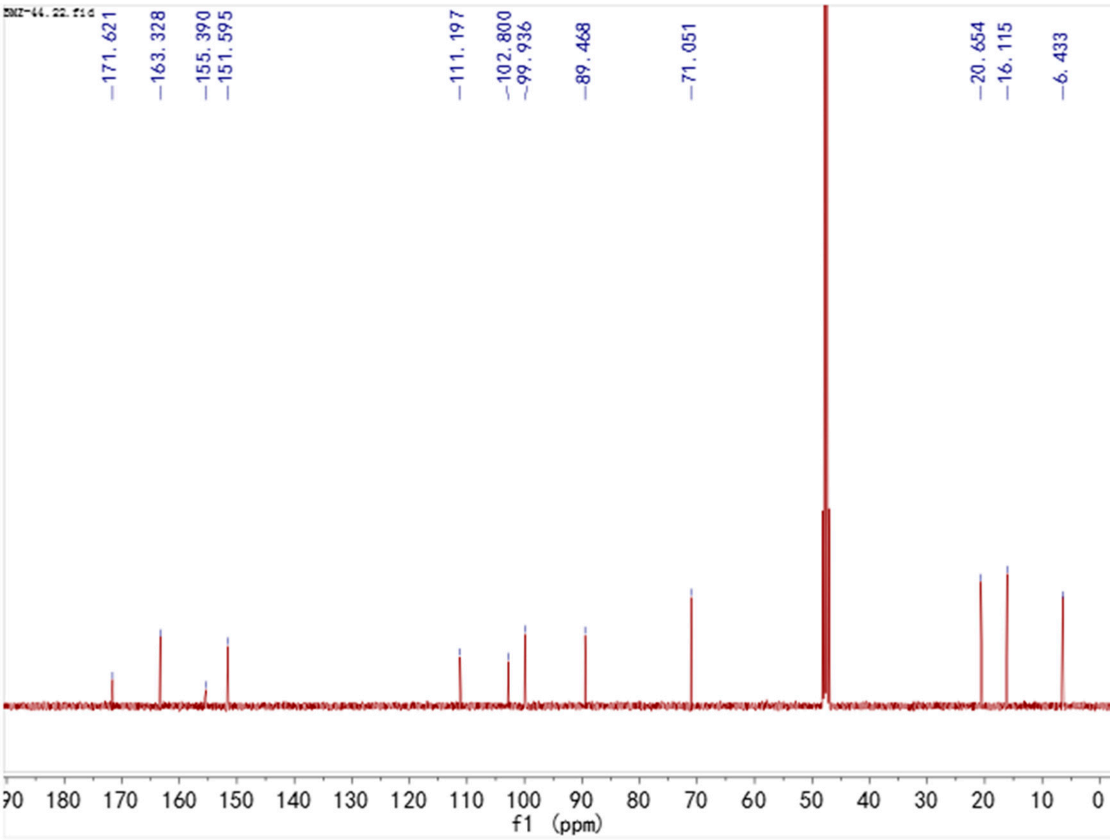

Figure S3. ^{13}C NMR spectrum of compound **1**.

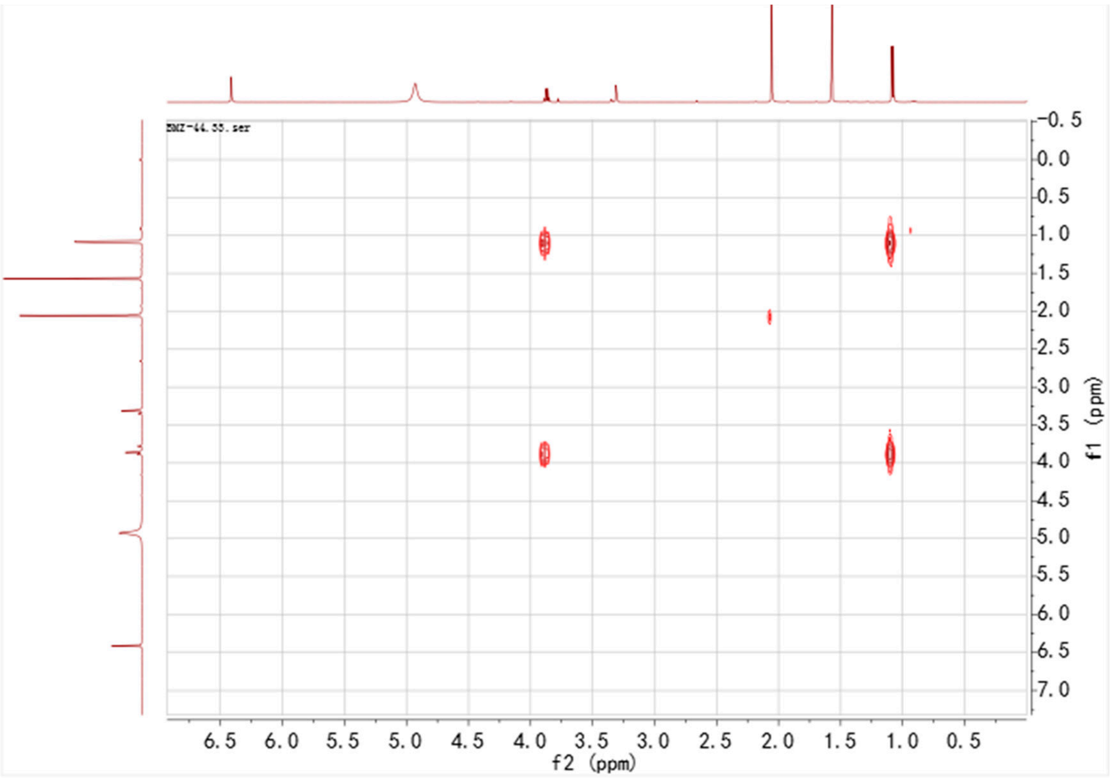

Figure S4. ^{1}H-^{1}H COSY spectrum of compound **1**.

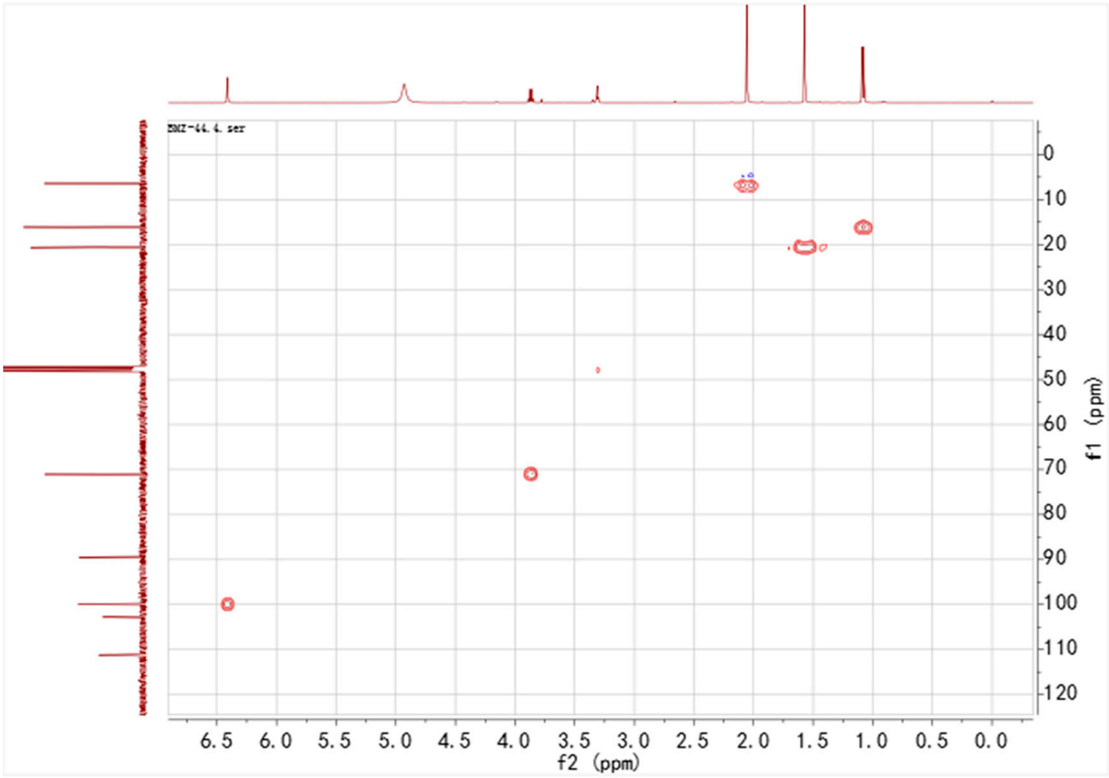

Figure S5. HSQC spectrum of compound **1**.

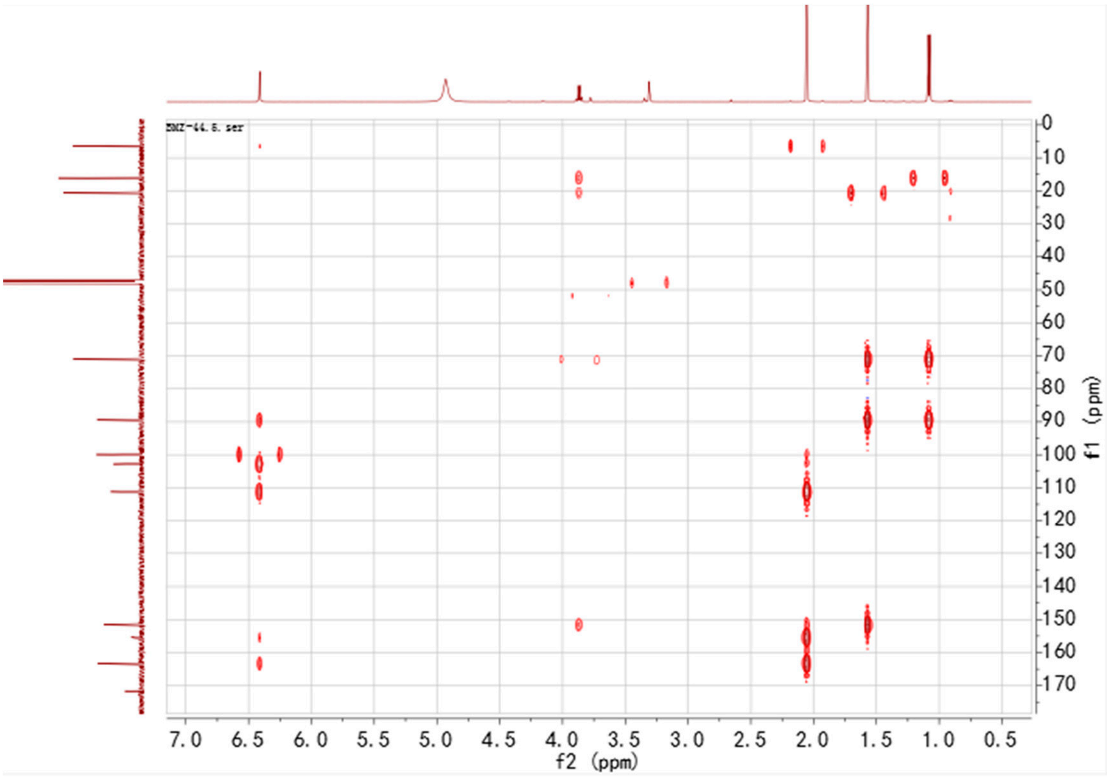

Figure S6. HMBC spectrum of compound **1**.

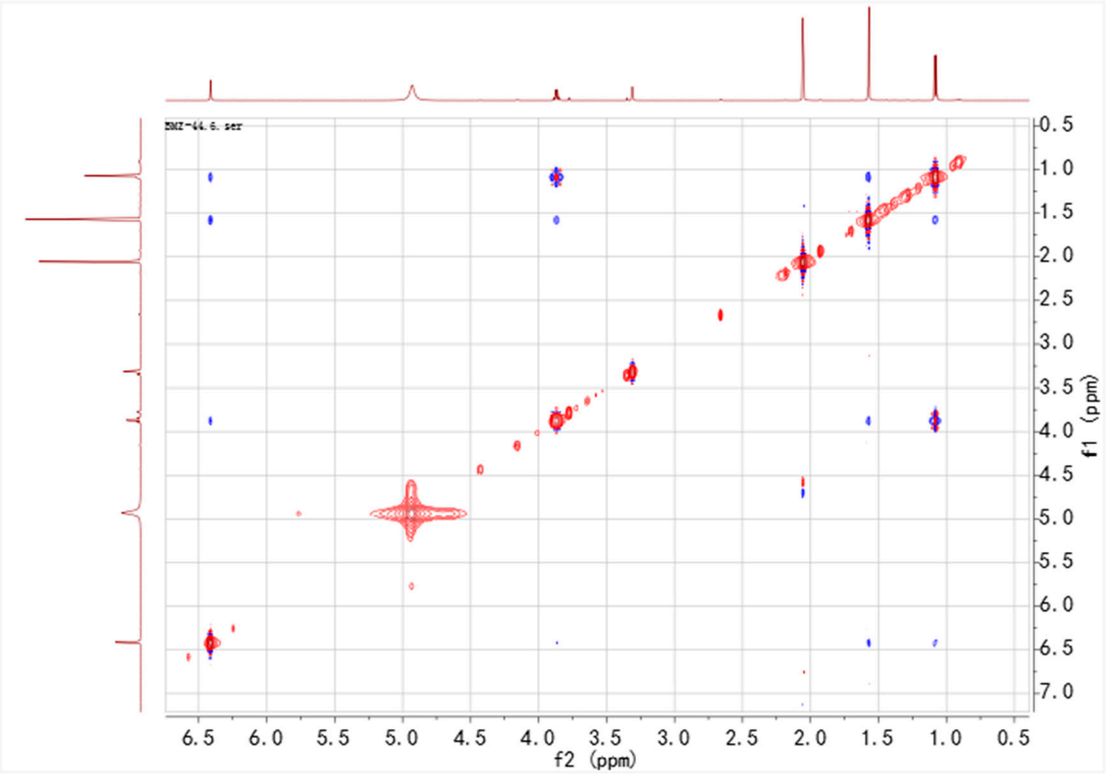

Figure S7. NOESY spectrum of compound **1**.

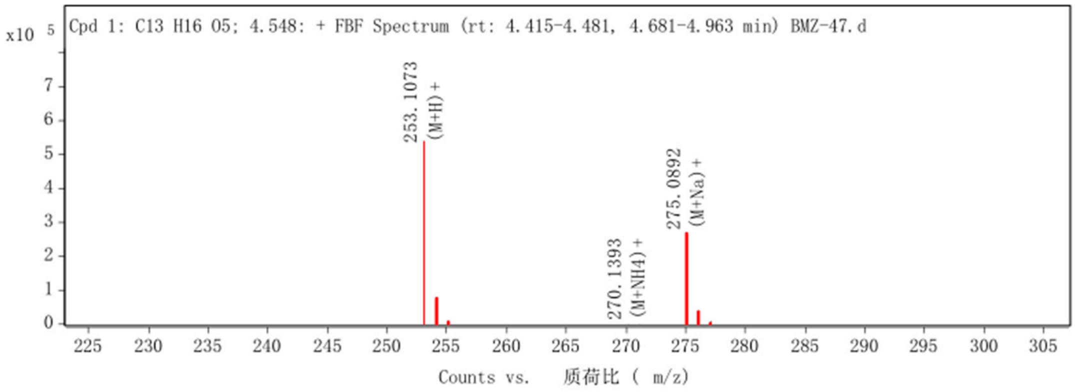

Figure S8. HRESIMS spectrum of compound **2**.

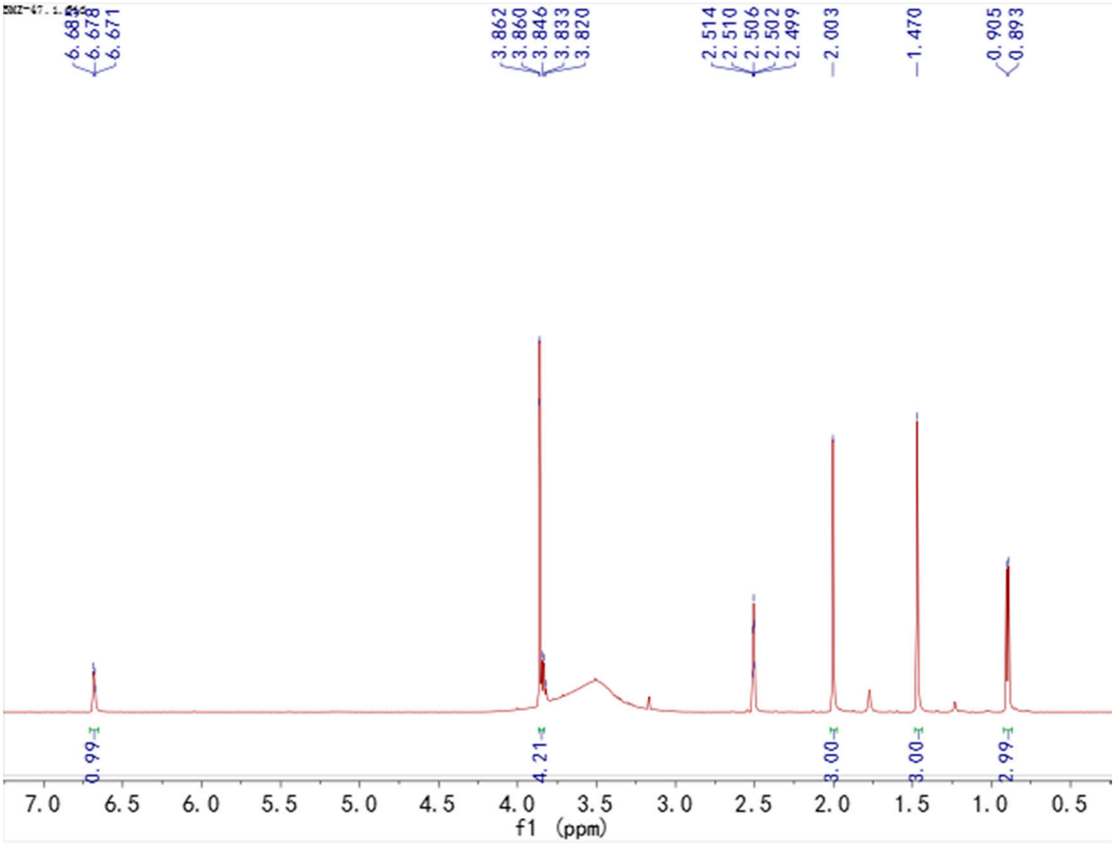

Figure S9. ^{1}H NMR spectrum of compound **2**.

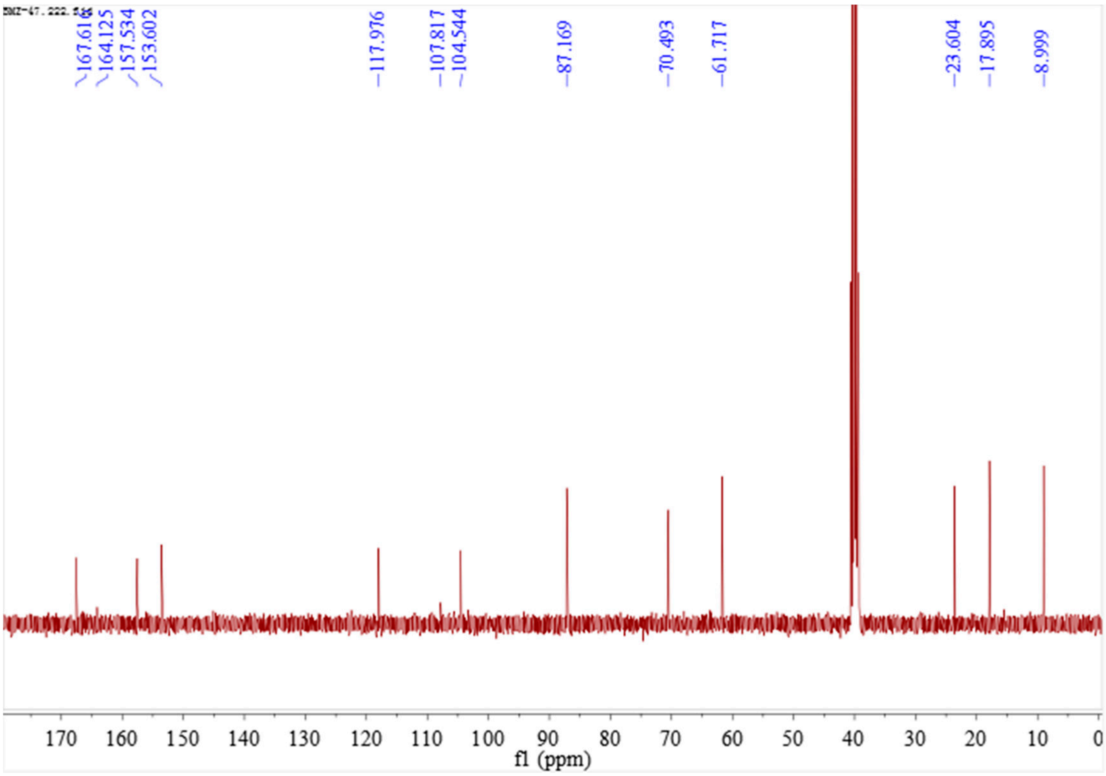

Figure S10. ^{13}C NMR spectrum of compound **2**.

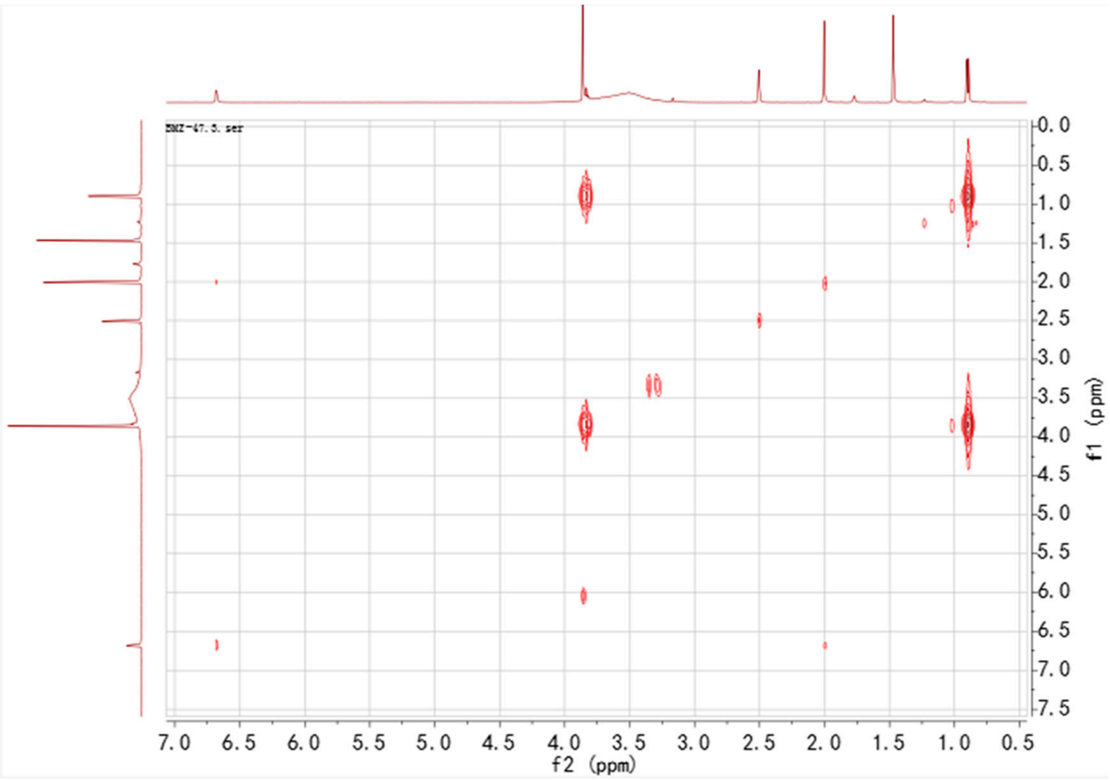

Figure S11. ^{1}H-^{1}H COSY spectrum of compound **2**.

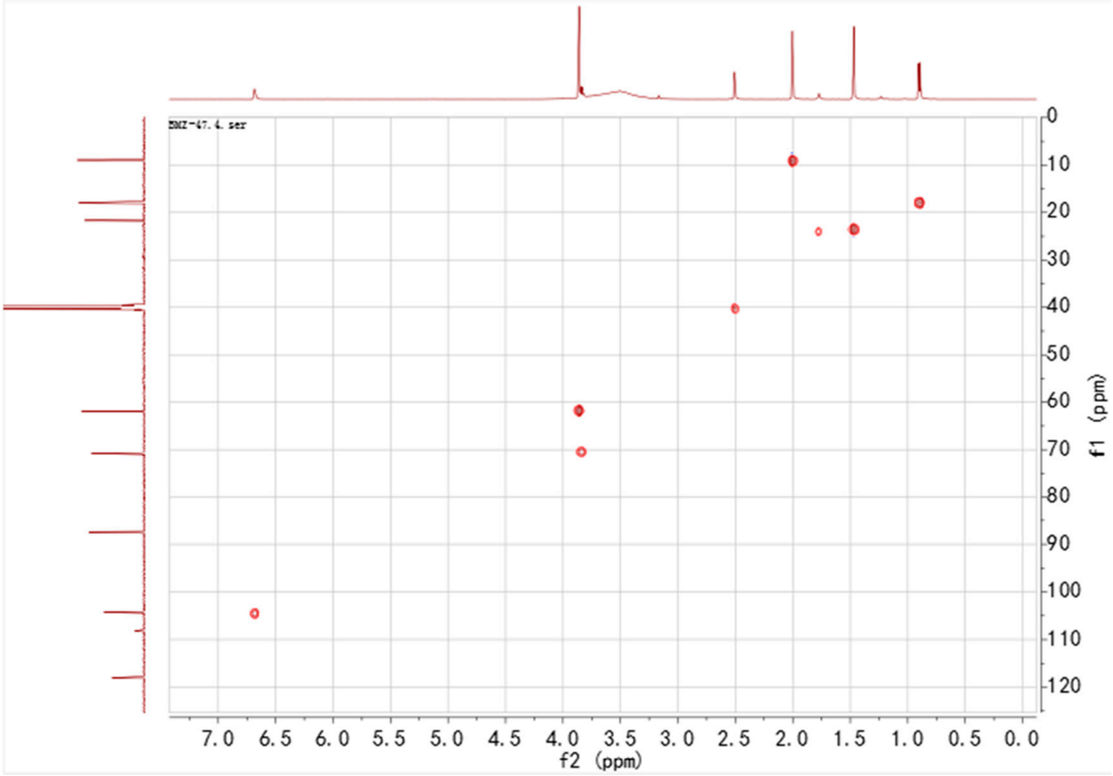

Figure S12. HSQC spectrum of compound **2**.

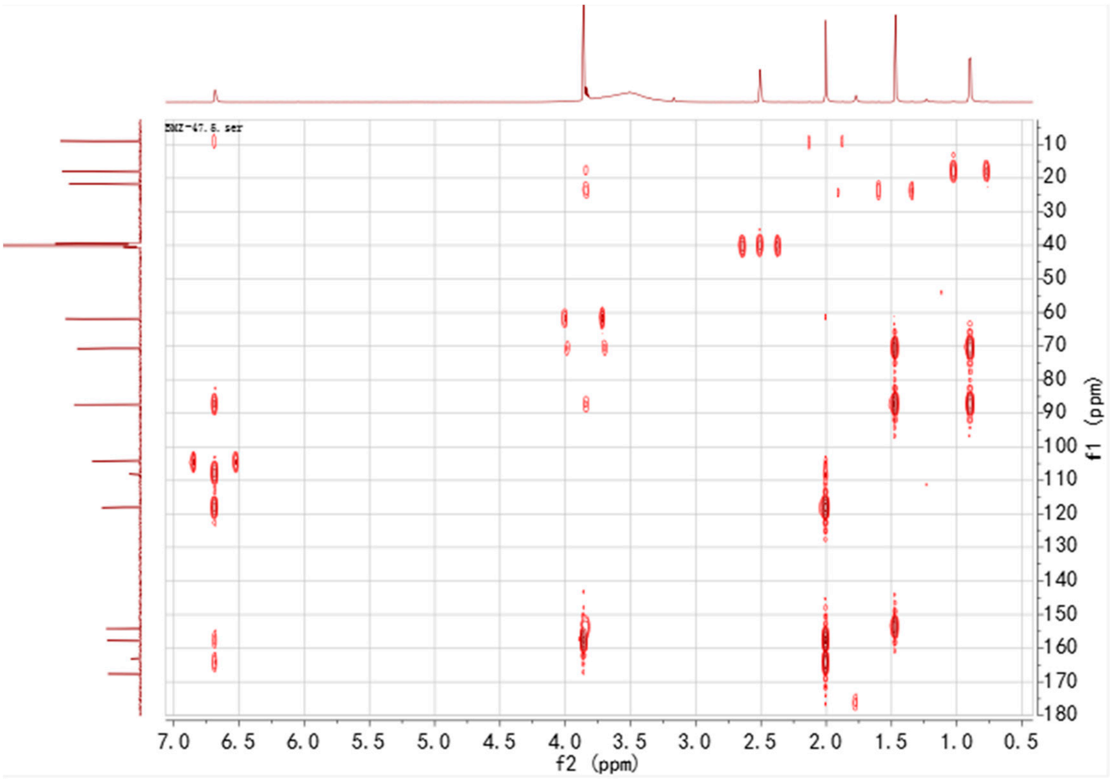

Figure S13. HMBC spectrum of compound **2**.

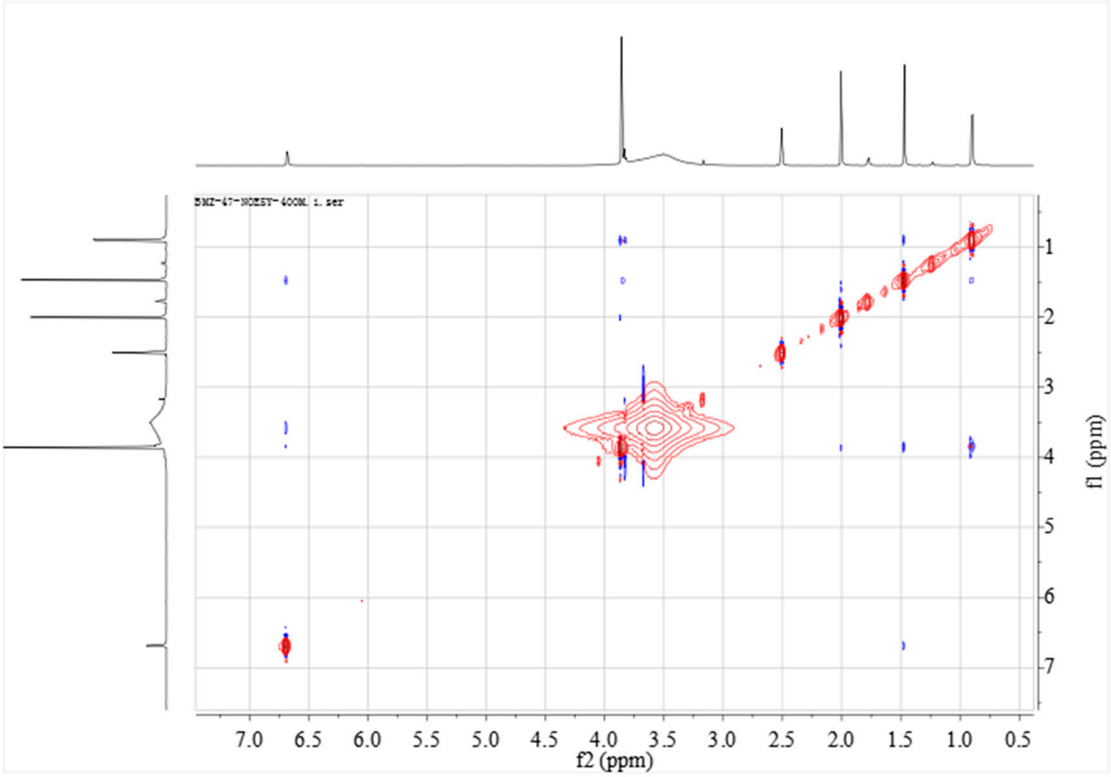

Figure S14. NOESY spectrum of compound **2**.

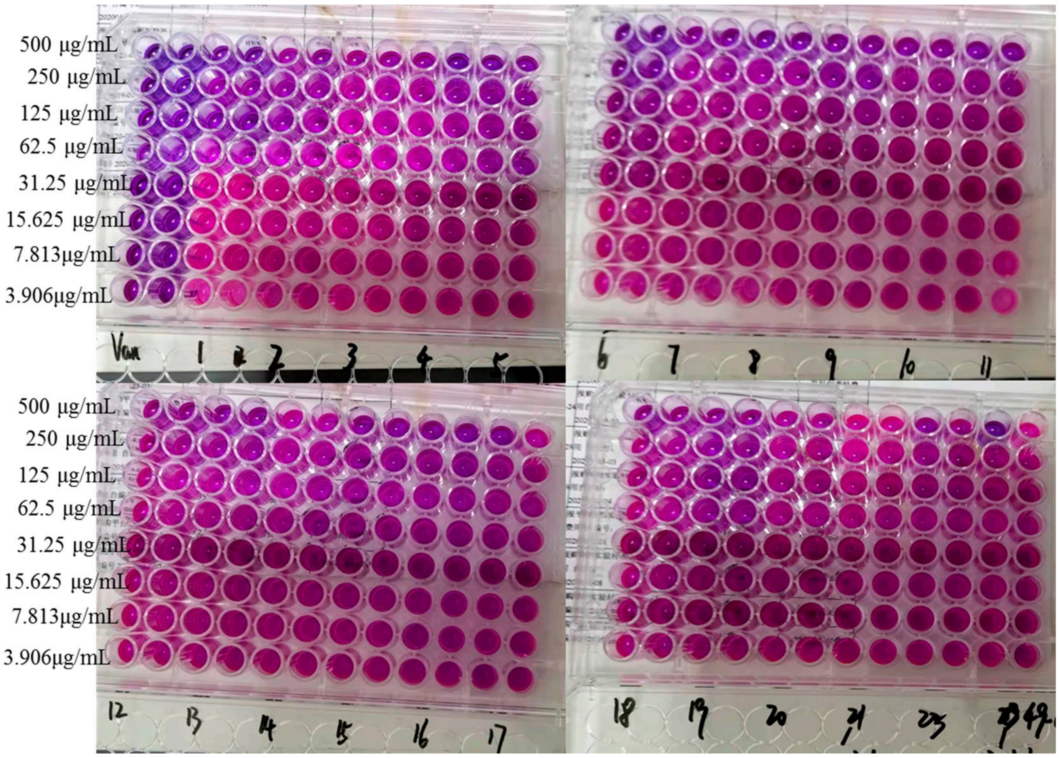

Figure S15. Antimicrobial activity assay results. (Numbers 10-19 in the figure are compounds **3**-**12**, numbers 20-21 are compounds **1**-**2**, and "van" is positive control vancomycin)

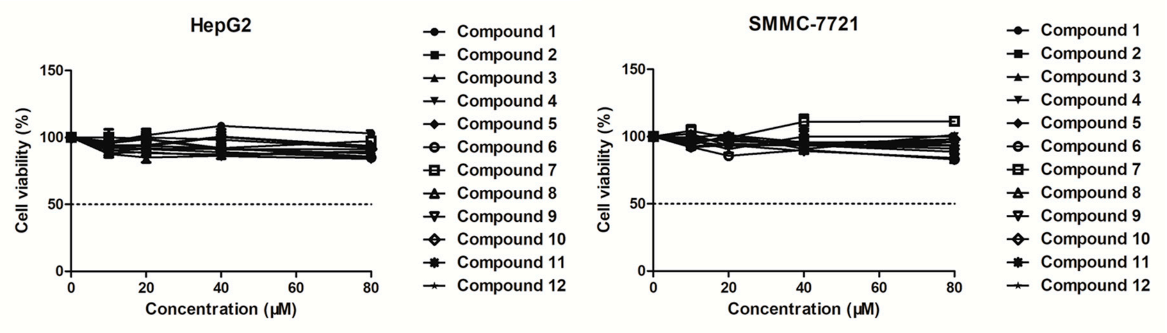

Figure S16. Dose-response curves for human hepatoma cell lines (HepG2 and SMMC7721).
